# Triple‐Helix Biomolecules Construct Self‐Adaptive Multi‐Phase Interfaces for Dendrites Immunized Zinc Metal Anodes

**DOI:** 10.1002/advs.77079

**Published:** 2026-08-12

**Authors:** Tianyi Wang, Jiahui Lu, Wei Gu, Jianfei Shi, Yuting Qin, Jian Yang, Di He, Zixia Lin, Jiabao Li, Bing Sun, Guoxiu Wang, Chengyin Wang

**Affiliations:** ^1^ School of Chemistry and Materials Yangzhou University Yangzhou Jiangsu Province People's Republic of China; ^2^ Guangxi University Nanning Guangxi Province People's Republic of China; ^3^ College of Chemistry and Chemical Engineering, Key Lab of Fluorine and Silicon for Energy Materials and Chemistry of Ministry of Education Jiangxi Normal University Nanchang Jiangxi Province People's Republic of China; ^4^ Test Center of Yangzhou University Yangzhou Jiangsu Province People's Republic of China; ^5^ Centre For Clean Energy Technology, School of Mathematical and Physical Sciences, Faculty of Science University of Technology Sydney Sydney NSW Australia

**Keywords:** aqueous batteries, proteins, separators, triple‐helix biomolecules, zinc metal anode

## Abstract

Aqueous zinc (Zn) metal cells are promising candidates for large‐scale energy storage owing to their intrinsic safety and low cost. However, their practical application remains limited by uncontrolled Zn dendrite growth and hydrogen‐evolution‐induced corrosion. Here, we present a collagen‐engineered strategy that harnesses the natural triple‐helix architecture of collagen to construct collagen‐coated cotton fiber (COL@COT) separators capable of continuous collagen release and dynamic interfacial regulation. The ordered triple‐helix chains enable directional Zn^2+^ migration through interconnected ion channels bridging the anode, electrolyte, and cathode, while the released collagen molecules migrate under the electric field to assemble a self‐adaptive organic‐inorganic SEI. During prolonged cycling, inner layer collagen undergoes a secondary‐structure evolution from the triple‐helix to β‐sheet domains, giving rise to a multilayered SEI with both flexibility and rigidity that maintains long‐term interfacial stability. This adaptive interphase lowers Zn‐ion desolvation barriers, directs (002)‐oriented Zn deposition, and suppresses water‐induced side reactions, thereby ensuring dendrite‐free and durable Zn plating/stripping. As a result, Zn||Zn symmetric cells exhibit ultralong cycling stability over 2200 h at 5 mA cm^−2^ and 1 mAh cm^−2^. This work introduces a secondary‐structure–programmed biomolecular approach for interphase engineering, bridging structural biology and electrochemistry to inspire the design of sustainable, bio‐derived energy storage systems.

## Graphical abstract

A bio‐derived triple‐helix framework enables adaptive interphase engineering for aqueous Zn metal batteries. Directional Zn^2^^+^ migration channels and dynamic transformation into β‐sheet domains construct an optimized solid electrolyte interphase with flexible–rigid synergy, delivering dendrite‐free Zn deposition and exceptional long‐term battery cycling stability.

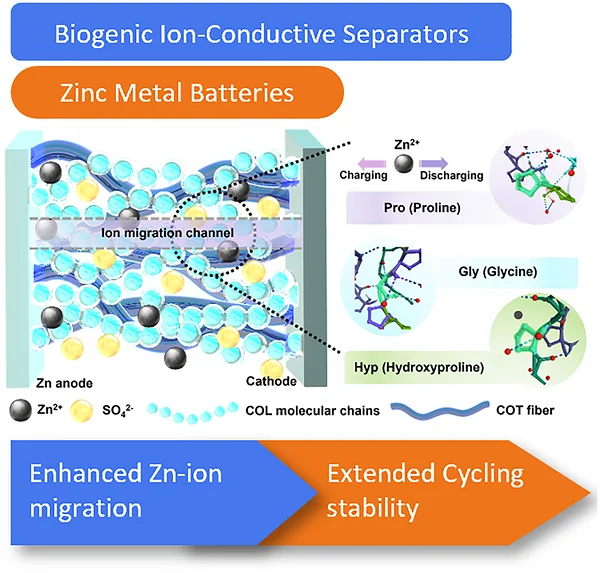

## 1 Introduction

Aqueous zinc metal batteries (AZMBs) have attracted increasing attention as next‐generation energy storage systems owing to their intrinsic safety, low cost, high theoretical capacity (820 mAh g^−1^), and low redox potential (‐0.76 V vs. SHE) [1, 2]. With non‐flammable aqueous electrolytes and earth‐abundant Zn anodes, AZMBs offer advantages over lithium‐ and sodium‐ion batteries in sustainability and safety [3, 4, 5, 6]. Nevertheless, uncontrollable Zn dendrite growth, hydrogen evolution, and surface corrosion severely compromise reversibility and stability, underscoring the urgent need for advanced interfacial engineering strategies [7, 8]. To mitigate these issues, electrolyte optimization, artificial solid electrolyte interphase (SEI) construction, and multifunctional protective coatings have been investigated [9, 10]. Among them, SEI engineering has proven particularly effective in homogenizing Zn deposition and suppressing side reactions, thereby extending Zn anode lifespan [11, 12]. Recent zincophilic‐site engineering has demonstrated that functional interfacial layers on Zn metal can regulate Zn nucleation, homogenize Zn^2^^+^ flux, and suppress water‐induced side reactions. For example, a 3D TiO_2_/Cu_2_Se/C zincophilic and hydrophobic interphase buffer layer was recently developed by integrating abundant Zn nucleation sites, built‐in electric‐field regulation, and hydrophobic protection against corrosion and hydrogen evolution. In parallel, advances in functional binders and aqueous polymeric components have highlighted the importance of mechanical integrity, interfacial adhesion, ion/electron transport, and chemical capture capability for practical battery interfaces [13, 14, 15, 16]. Nevertheless, most direct Zn‐surface coatings and synthetic polymeric components are essentially static once fabricated, limiting their ability to adapt to the continuously evolving Zn plating/stripping interface. This limitation motivates the development of sustainable, bio‐derived, and structurally programmable interfacial regulators capable of dynamic participation in SEI construction [17, 18, 19].

Biomaterials have recently emerged as attractive alternatives due to their renewability, biodegradability, and structural diversity [20]. Their dynamic interactions with Zn^2+^ can yield adaptive interphases that stabilize metal electrodes. For instance, Wu et al. reported that multifunctional cellulose nanocrystal additives can effectively improve the surficial stability of Zn metal anodes [21]. Similarly, Chen et al. reported that a natural polysaccharide‐based coating effectively regulated Zn^2+^ flux and suppressed dendrite formation, achieving long‐term cycling stability [22]. Despite these advances, proteins stand out by combining ion selectivity with structural complexity, enabling precise interfacial regulation. Proteins inherently bind Zn^2+^, facilitating desolvation and uniform deposition [23, 24, 25], while their amphiphilic nature and abundant functional groups make them versatile for adaptive SEI construction [26, 27]. Recently, collagen‐mediated electrolyte regulation has also been reported to reconstruct the Zn^2+^ solvation sheath, promote preferential Zn(002) deposition, and induce a ZnF_2_/Zn_3_N_2_‐rich SEI for high‐rate Zn anodes [28]. In our previous studies, silk fibroin (SF) additives and bovine serum albumin (BSA)‐based bifunctional coatings have proven that specific proteins can effectively stabilize Zn anodes, achieving cycling lifetimes of up to 2400 h [29, 30, 31]. Distinct from these previously reported protein systems, collagen possesses a unique Gly–X–Y sequence‐defined triple‐helix architecture rather than β‐sheet‐rich or globular conformations. This ordered molecular framework spatially arranges abundant O/N‐containing coordination sites along robust helical chains, providing both Zn^2^^+^‐binding capability and high‐flux ion‐transport pathways. Moreover, the present COL@COT separator differs from directly dissolved protein additives or static protein coatings by serving as a sustained‐release biomolecular reservoir, enabling continuous collagen release, electric‐field‐driven migration, and dynamic participation in SEI construction during cycling. These structural and functional distinctions make collagen a programmable platform for self‐adaptive SEI engineering. These results highlight proteins as promising interfacial regulators.

Nevertheless, the potential of protein‐based materials in self‐adaptive SEI engineering remains largely underexplored [23, 32]. Triple‐helical proteins, such as collagen (COL), a structural protein derived from animal connective tissues, is particularly compelling owing to its unique triple‐helix conformation—a complex molecular architecture that remains extremely difficult to reproduce synthetically [33]. Its amino acid constituents (e.g., glycine (Gly), proline (Pro), and hydroxyproline (Hyp)) provide abundant coordination sites for Zn^2+^ binding and transport [34], thereby regulating local ion concentration and associated biological functions such as enzymatic catalysis and signal transduction [35]. Furthermore, the ordered triple‐helix arrangement confers high mechanical robustness and biostability, enabling the assembly of well‐defined frameworks with inherently high‐flux ion‐conducting channels [36]. These attributes make collagen a promising biomolecular platform for constructing adaptive SEIs that suppress dendrites, mitigate parasitic reactions, and stabilize Zn metal anodes.

In this work, as demonstrated in Figure 1a, to maintain a stable and relatively low concentration of COL in the electrolyte, COL was immobilized onto the surface of cotton fiber (COT), thereby constructing a COL@COT separator with sustained‐release functionality. Compared with traditional glass fiber (GF) separator, COL@COT enables dynamic regulation of interfacial chemistry at Zn deposition sites, thereby achieving ultralong cycling stability of Zn anodes. Unlike conventional inert substrates, this separator serves as a reservoir for collagen molecules that can be slowly released into the electrolyte. The isoelectric point (pI) refers to the pH at which a protein carries no net electrical charge. When the solution pH is lower than the pI, protonation of ionizable groups, such as amino and carboxyl‐related residues, gives the protein an overall positive charge; when the solution pH is higher than the pI, the protein generally becomes negatively charged. The pI of type II collagen is approximately 7.2, whereas the pH of the 2 M ZnSO_4_ electrolyte is around 4.3. Therefore, collagen molecules released from the COL@COT separator are positively charged in the electrolyte and can migrate toward the negatively polarized Zn electrode under the electric field during Zn deposition. This electric‐field‐driven migration enables collagen molecules to participate dynamically in interfacial SEI formation at the Zn surface. Compared with conventional double‐helix or single‐helix biomolecular frameworks, these triple‐helix channels exhibit higher ion flux and superior structural stability, thereby providing more durable suppression of dendrite growth and mitigation of side reactions. Moreover, migrating collagen molecules participate in the formation of a stable self‐adaptive organic–inorganic SEI, in which organic (COL) and inorganic (ZnSO_4_) components synergistically stabilize the electrode. This adaptive interface lowers Zn^2+^ desolvation barriers, suppresses parasitic reactions, and establishes a continuous Zn‐ion transport pathway across the electrolyte. Benefiting from these synergistic effects, Zn||Zn symmetric cells with the COL@COT separator exhibit an ultralong lifespan exceeding 2200 h at 5 mA cm^−2^ and 1 mAh cm^−2^, nearly 16 times longer than those with GF separators. Furthermore, excellent cycling stability is also achieved in Zn||Cu half‐cells and Zn||V_2_O_5_ full cells, highlighting the broad applicability and superior protective performance of this biomolecule‐engineered separator in aqueous Zn metal cells.

**FIGURE 1:**
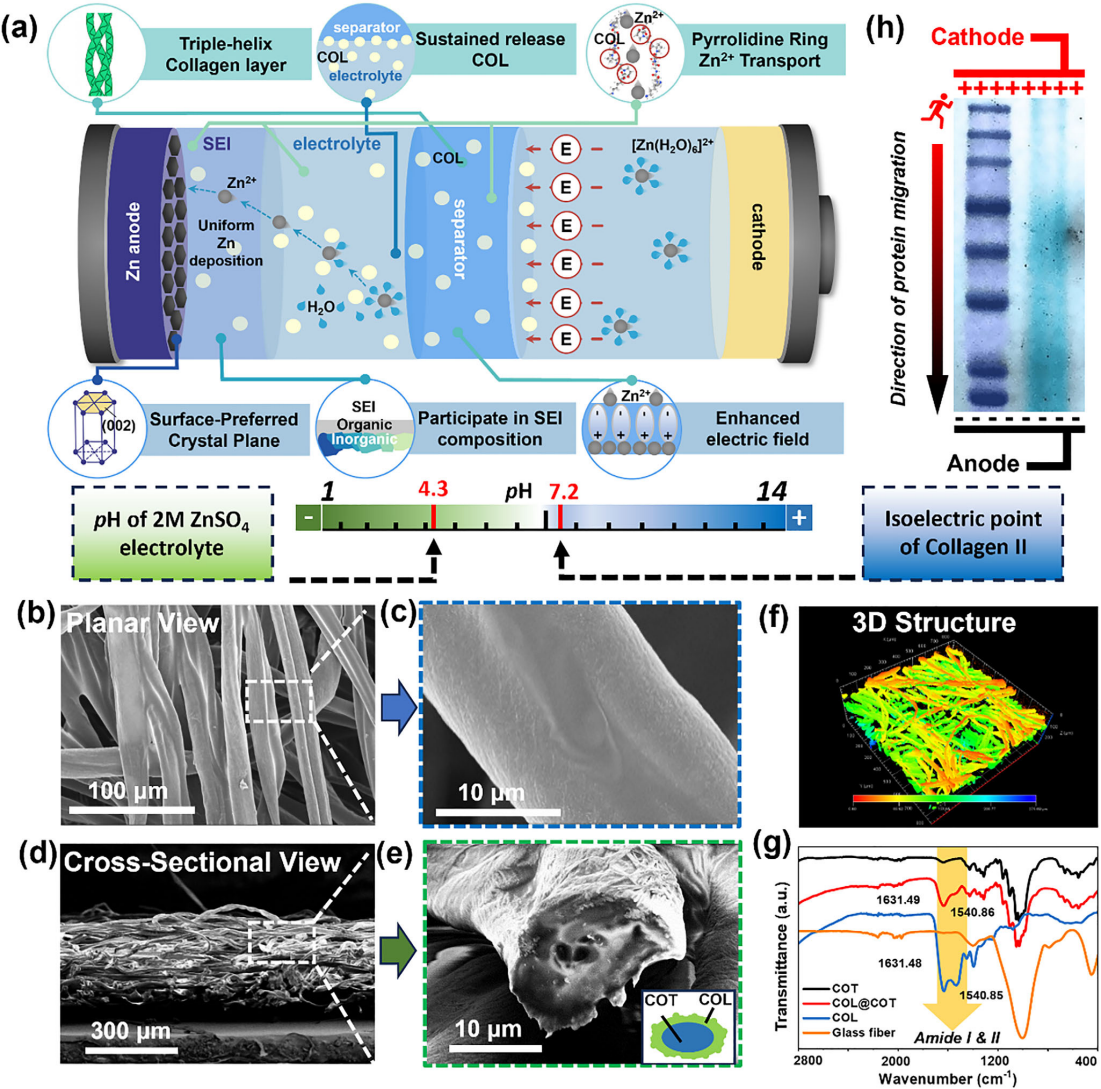
(a) Schematic illustration of the advanced features of the COL@COT separator for AZMBs and the electric‐field‐driven migration of collagen molecules. SEM images of the (b, c) surface and (d, e) cross‐sectional images of a COL@COT separator and fiber. (f) Two‐photon confocal microscopy image of a COL@COT separator. (g) FT‐IR spectrum of COT, COL, COL@COT, and GF. (h) The Native‐PAGE result displays the migration trace of COL molecules in the electrolyte.

## 2 Results and Discussion

To evaluate the structural characteristics and cost‐effectiveness of different separators, digital image analysis was performed on COT, COL@COT, and their morphologies after immersion in ZnSO_4_ electrolyte. As shown in Figure S1, unit‐area cost analysis indicates that GF is the highest, while COT is the most economical. The unpunched COT separator exhibits a uniform structure. Morphological comparisons between COT and COL@COT reveal that COL@COT displays a more homogeneous texture than pristine COT, and noticeable surface changes appear after immersion in 2 M ZnSO_4_ electrolyte. The surface and cross‐sectional morphologies were further examined by SEM. As demonstrated in Figure S2, GF consists of evenly distributed fibers with an average diameter of 0.94 ± 0.62 µm, whereas COT fibers are considerably thicker and more heterogeneous (12.94 ± 3.96 µm, Figure S3). In contrast, COL@COT fibers have the highest diameter (Figure 1b,c), but are significantly more uniform (20.25 ± 1.78 µm), contributing to improved porosity and electrolyte wettability. The smoother surface of COL@COT compared with unmodified COT ensures more uniform ion distribution.

Electrolyte infiltration tests (Figure S4) confirmed the superior wettability of COL@COT: complete electrolyte absorption occurred within 0.3 s, while COT and GF retained contact angles of 41.4° and 60.7°, respectively. The cross‐sectional SEM image of the COL@COT separator (Figure 1d) reveals a fluffy and highly porous architecture with a thickness of approximately 300 µm. This self‐supporting structure ensures excellent mechanical integrity and intimate interfacial contact with the Zn metal anode during prolonged cycling. As shown in Figure 1e, the cross‐sectional view of an individual COL@COT fiber clearly displays a well‐defined outer amorphous COL coating layer surrounding a dense COL‐based inner core, herein accounting for the high electrolyte wettability of COL@COT separator. To further evaluate the surface topography and distribution uniformity of the collagen‐modified separator, Kelvin probe force microscopy (KPFM) characterization was performed. As shown in Figure S5, pristine COL exhibits an uneven surface with pronounced height fluctuations. In contrast, COL@COT shows a smoother and more continuous surface morphology, supporting the formation of a homogeneous collagen‐modified fiber surface. The corresponding KPFM topographic analysis further confirms the improved surface uniformity of COL@COT, which is consistent with the cross‐sectional SEM and two‐photon confocal fluorescence microscopy results. The two‐photon confocal fluorescence microscopy further revealed a continuous and uniform protein coating on COL@COT fibers. As shown in Figure 1f and Figure S6), in sharp contrast to the negligible fluorescence of pristine COT, three‐dimensional reconstructions verified the uniform spatial distribution of the protein layer, which enhances separator wettability and ion transport.

The successful coating of COL on COT was further confirmed by Fourier‐transform infrared spectroscopy (FT‐IR). As shown in Figure 1g, COL@COT exhibits characteristic protein peaks at 1631.4 and 1540.8 cm^−1^, corresponding to the amide I band (C═O stretching vibration) and *Amide II* band (N─H bending and C─N stretching vibrations), respectively. In addition, we evaluated the structural stability of COL after cell cycling by analyzing the changes in COL's secondary structure. As depicted in Figure S7, after being attached to the COT fibers, the secondary structure of collagen remained unchanged, exhibiting the same triple‐helix configuration as the native COL molecules. As shown in Figure 1h, the Native Polyacrylamide Gel Electrophoresis (Native‐PAGE) testing results successfully captured the migration trajectory of COL molecules towards the Zn anode driven by the electric field (marked by bromothymol blue), in which COL protein migrated from the cathode electrode (upper end) toward the anode electrode (lower end). The electrophoresis results displayed a series of distinct protein bands moving from the sample well (cathode electrode) downwards to the anode electrode. This indicates that COL protein can be released from the separator and migrate toward the anode electrode under an electric field, which is consistent with the hypothesis supposed above.

To further investigate the distribution and movement of COL on the Zn metal anode, we conducted fluorescence/luminescence and two‐photon confocal microscopy for labelling and observation [29, 37]. The defective Zn foils, retrieved from both the COL@COT‐equipped Zn||Zn symmetric cell and the cell containing COL‐added electrolyte, were washed twice with deionized water and subsequently examined under ultraviolet (UV) light. As shown in Figure S8, it is evident that the Zn anode employing the COL@COT separator exhibits a markedly higher surface loading of COL than the counterpart using COL solely as an electrolyte additive. Moreover, even after being washed with deionized water, the COL fluorescence signal remains significantly stronger at high‐curvature regions (e.g., defect edges) than at flatter, low‐curvature sites. This phenomenon can be attributed not only to the intensified local electric field at high‐curvature sites that enhances the electrostatic attraction of COL molecules, but also, more importantly, to the preferential accumulation of protein molecules at these regions driven by positive cooperative effects.

In view of the above results, as demonstrated in Figure 2a, we herein propose a novel bio‐self‐assembly interfacial protection mechanism. Upon release from the COL@COT substrate, COL molecules carry positive charges and migrate toward the Zn anode under the electric field. The early‐arriving COL molecules undergo a secondary structural transition at the nascent Zn surface, transforming from the native triple helix into β‐sheet domains (SEI *I*). Compared with the initial triple‐helix conformation, the β‐sheet structure possesses a higher packing density and greater mechanical robustness, while the unfolding of the triple helix also exposes additional hydrophobic groups. These combined features not only suppress the activity of interfacial water molecules but also accommodate the stress fluctuations arising from Zn deposition. In contrast, the subsequently arriving COL molecules largely retain the triple‐helix structure (SEI *II*). Importantly, the interior of the triple helix contains abundant amino acid motifs (e.g., Gly, Pro, Hyp) capable of chelating Zn^2+^ ions, thereby creating efficient ion‐transport channels at the interface. Moreover, the peripheral polar groups of COL assemble through hydrogen bonding into a crosslinked network, leading to the formation of a three‐dimensional, ordered, biphasic bio‐interfacial SEI layer that effectively blocks solvent penetration.

**FIGURE 2:**
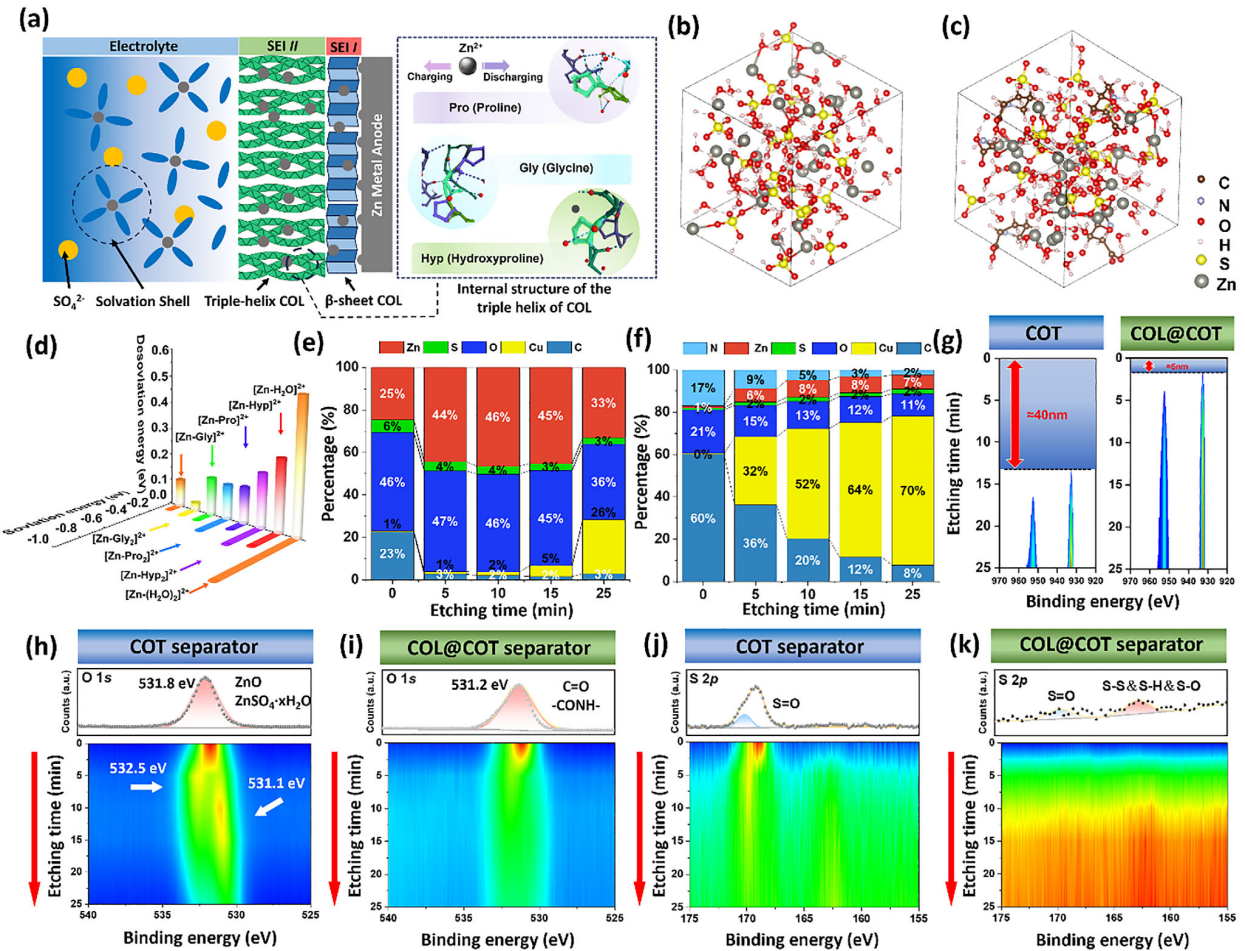
(a) Schematic illustration of the ion migration pathway in aqueous Zn batteries with COL@COT. b, c) Solvation structures of Zn‐ion in (b) blank ZnSO_4_ electrolyte and (c) ZnSO_4_ electrolyte with COL based on AIMD simulations. (d) Solvation and desolvation energies of a Zn‐ion with water molecules and three representative amino acids from COL (Gly, Pro, Hyp). (e, f) XPS depth analysis of the SEI layer on Cu foil from Zn||Cu cells with COL@COT and COT separators, including (e, f) element ratio survey, g) Cu 2p, (h, i) O 1s, and (j, k) S 2p spectra.

To investigate the interactions between COL and Zn^2^^+^, ab initio molecular dynamics (AIMD) simulations were performed to probe the changes in ZnSO_4_ solvation structures after introducing representative collagen residues, including Gly, Pro, and Hyp [38]. Snapshots of the solvation environment with and without additives are presented in Figure 2b,c. In the pristine ZnSO_4_ electrolyte (Figure 2b), Zn^2+^ is predominantly coordinated by oxygen atoms from water molecules, forming a regular octahedral geometry with a coordination number of six. This confirms that water molecules dominate the solvation shell in aqueous ZnSO_4_. In contrast, when COL is present (Figure 2c), the Zn^2^^+^ coordination environment is remodeled mainly through interactions with oxygen‐ and nitrogen‐containing groups in collagen residues, together with water‐derived oxygen atoms. Notably, because the representative collagen residues used in the AIMD simulations, including Gly, Pro, and Hyp, do not contain sulfur atoms, the Zn–S correlation in the simulation should be assigned to sulfate sulfur from the ZnSO_4_ electrolyte rather than to sulfur‐containing groups from collagen. Such competitive coordination substantially remodels the coordination environment of Zn^2^^+^ and diminishes the dominant role of water molecules in the primary solvation sheath. Beyond structural perturbations, COL exerts a decisive influence on Zn^2^^+^ desolvation, a critical step controlling Zn deposition kinetics. At the molecular scale, desolvation requires the release of water molecules from the solvation shell before Zn^2^^+^ can migrate to the electrode surface. The incorporation of COL residues partially replaces coordinated water molecules through Zn^2^^+^–O/N interactions, establishing stable coordination with Zn^2^^+^. This replacement lowers the desolvation energy barrier, thereby facilitating Zn^2^^+^ transfer into the solid phase. Moreover, by limiting the retention of water in the solvation sheath and weakening Zn^2^^+^–SO_4_^2^^−^ association, COL accelerates Zn^2^^+^ transport and promotes faster deposition kinetics.

The impact of COL on the Zn^2^^+^ solvation environment was further investigated by radial distribution functions (g(r)) of Zn–S and Zn–O [39, 40, 41]. As shown in Figure S9, in the pristine ZnSO_4_ electrolyte, the Zn–S g(r) shows a prominent peak at around 3.05 Å, which reflects the spatial correlation between Zn^2^^+^ and sulfate sulfur in SO_4_^2^^−^ anions. After introducing representative collagen residues, the Zn–S peak intensity decreases, and the peak position shifts slightly, indicating that COL weakens the association between Zn^2^^+^ and SO_4_^2^^−^ rather than providing direct sulfur coordination sites. Among the representative collagen residues, Pro shows the most pronounced effect, reducing the Zn–S peak intensity from 3.15 to 1.90 and shifting the peak position from 3.05 to 3.33 Å, suggesting a substantially weakened Zn^2^^+^–SO_4_^2^^−^ ion‐pairing interaction. This effect can be attributed to competitive coordination between Zn^2^^+^ and the O/N‐containing groups of collagen residues, which perturbs Zn^2^^+^–SO_4_^2^^−^ association and remodels the Zn^2^^+^ solvation structure. Consistently, the Zn–O g(r) also changes after COL introduction. In particular, Pro reduces the Zn–O peak intensity by 0.72 and shifts the peak position by 0.14 Å, further confirming the participation of collagen‐derived oxygen‐containing groups in Zn^2^^+^ coordination. These results demonstrate that COL regulates the Zn^2^^+^ solvation environment mainly through O/N coordination and by weakening Zn^2^^+^–SO_4_^2^^−^ association, thereby facilitating Zn^2^^+^ desolvation and interfacial transport.

In aqueous Zn cells, Zn^2+^ desolvation energy critically governs the deposition process, as it represents the barrier that must be overcome for Zn^2+^ to shed its hydration shell and migrate to the electrode surface. In aqueous solution, Zn^2+^ typically exists as [Zn (H_2_O)_6_]^2+^, and desolvation requires breaking Zn–O coordination bonds, which is energetically demanding [42, 43]. To quantify the effect of COL, we calculated the desolvation energies of Zn^2+^ coordinated with H_2_O and three representative amino acids (Gly, Pro, Hyp) using molecular models (Figure S10). For [Zn (H_2_O)_2_]^2+^, the desolvation energy is 0.504 eV, whereas Zn^2+^ coordinated with Gly, Pro, and Hyp exhibits much lower values of 0.029, 0.131, and 0.205 eV, respectively (Figure 2d). These results demonstrate that Zn^2+^ is far more easily released from Zn^2^^+^–O/N interactions than from water. The reduced energy barrier arises from the amino and carboxyl groups of COL, which establish stable yet labile coordination with Zn^2+^.This effect is particularly pronounced for Gly, whose simple structure and strong coordination affinity result in the lowest desolvation energy. By facilitating Zn^2+^ release, COL not only remodels the solvation structure but also accelerates Zn^2+^ transport and deposition kinetics. Consequently, the presence of COL residues—especially Gly—promotes faster and more uniform Zn plating, in agreement with AIMD simulations showing partial replacement of water by Zn^2^^+^–O/N interactions. Overall, COL lowers the desolvation barrier and enhances local reaction kinetics, thereby improving the efficiency, stability, and reversibility of Zn metal anodes.

We performed X‐ray photoelectron spectroscopy (XPS) depth profiling to investigate the interfacial chemical evolution of cycled copper foils with different separators [44]. Prior to analysis, the electrodes were cycled for five cycles at a current density of 0.5 mA cm^−2^ with a capacity of 0.25 mAh cm^−2^. As shown in Figure 2e,f, the sample with a pure cotton (COT) separator exhibited only a carbon signal in the topmost layer, mainly arising from surface contamination due to detached carbon fibers. In contrast, the COL@COT separator produced an initial carbon content as high as 60%, accompanied by a markedly stronger nitrogen signal, confirming the strong adhesion of collagen molecules at the interface. Oxygen signals further highlight the difference between the two separators. For the COT separator, the oxygen content exceeded 40 % in the middle and upper layers, evidencing the accumulation of interfacial by‐products such as Zn (OH)_2_ and ZnSO_4_·xH_2_O. In sharp contrast, the COL@COT separator displayed a dominant O 1s peak centered at 531.2 eV in the topmost layer, originating from the carbonyl oxygen of protein amide groups (–CONH–) [45, 46]. The subsequent depth profile revealed a much weaker oxygen signal, demonstrating that collagen effectively suppressed parasitic side reactions. The difference in interphase thickness is evident from the copper signal onset (Figure 2g). With the COT separator, metallic Cu signals appeared only after 15 min of sputtering, whereas the COL@COT sample revealed Cu signals within 3 min. Considering the advanced zinc removal during etching, this result indicates that dead Zn and by‐products formed with COT are significantly thicker than those with COL@COT. To probe the interfacial chemistry of the deposited Zn metal, O 1s XPS depth profiling was conducted, with the results visualized as depth‐resolved contour plots. For the Zn anode cycled with the pristine COT separator (Figure 2h), distinct O 1s components at ∼532.5 and ∼531.1 eV is observed, corresponding to ZnO and hydrated zinc sulfate (ZnSO_4_·xH_2_O), respectively. Notably, the ZnSO_4_·xH_2_O signal progressively intensifies with increasing etching depth, indicating continuous accumulation of water‐induced byproducts within the deposited Zn layer. In contrast, the Zn anode regulated by the COL@COT separator exhibits a markedly simplified O 1s profile (Figure 2i), dominated by a single associated with collagen‐derived carbonyl species (C═O/–CONH–). No detectable ZnO or ZnSO_4_·xH_2_O signals are observed throughout the medium and lower depths, suggesting that COL effectively suppresses parasitic interfacial reactions and prevents the inward growth of inorganic byproducts, thereby establishing a chemically stable and organic‐dominated Zn interface.

Further evidence arises from the S 2p spectra (Figure 2j,k). With the COT separator, the strong S═O signal at 168.9 eV persists during etching, indicating the presence of sulfate‐containing byproducts, such as hydrated zinc sulfate species [47]. By contrast, the COL@COT sample exhibits a much weaker sulfate‐related signal, suggesting that COL suppresses the accumulation of sulfate‐rich byproducts at the Zn surface [48, 49, 50, 51]. The low‐binding‐energy sulfur feature observed after cycling is more reasonably associated with sulfate‐derived reduced sulfur species formed during interfacial reactions, rather than direct sulfur coordination from collagen. These results further support that COL regulates the Zn/electrolyte interface mainly by suppressing sulfate‐rich byproduct formation and remodeling the local Zn^2^^+^ coordination environment through O/N‐containing groups. Additional chemical changes induced by COL at the Zn surface are also evident from the C 1s, N 1s, and Zn 2p spectra (Figures S11–S13). Consistent with the thick by‐product‐rich interfacial layer observed by Cryo‐EM, the XPS depth‐profiling results reveal pronounced Zn–O/Zn–OH and sulfate‐related signals in the GF‐based cell, suggesting the accumulation of sulfate/hydroxide‐containing byproducts during cycling.

To elucidate the interfacial mechanism of COL on Zn metal anodes, we introduced a copper mesh into cells assembled with either GF or COL@COT separators and analyzed the SEI layers formed after Zn deposition using cryo‐electron microscopy (cryo‐EM, ‐185°C) under low‐dose imaging conditions [52, 53]. As shown in Figure 3a, the Zn surface in the cell with a conventional GF separator exhibits a well‐defined by‐product‐rich interfacial layer with a thickness approaching 100 nm. Because Cryo‐EM mainly provides morphological and thickness information, the specific chemical composition of this layer should not be determined from the Cryo‐EM image alone. Combined with the XPS depth‐profiling results, which reveal pronounced Zn–O/Zn–OH and sulfate‐related signals, this interfacial layer can be more cautiously described as a sulfate/hydroxide‐containing byproduct‐rich layer rather than being assigned to a specific crystalline phase. Moreover, distinct dendritic protrusions can be observed within this layer, indicating uncontrolled Zn nucleation and growth. In sharp contrast, the Zn surface protected by the COL@COT separator displays a much thinner SEI film of approximately 50 nm (Figure 3b). More importantly, this SEI exhibits a pronounced polymeric morphology, and the region within ∼15 nm adjacent to the Zn substrate shows a markedly higher density. To further clarify the chemical composition and spatial distribution of the COL‐regulated interfacial layer, TOF‐SIMS depth profiling was performed on the cycled Zn electrode with the COL@COT separator. As shown in Figure S14, the three‐dimensional reconstruction reveals pronounced collagen‐derived organic fragments, including CN^−^, CNO^−^, and the hydroxyproline‐related C_4_H_8_NO^+^ fragment, supporting the incorporation of collagen‐derived components into the SEI. Inorganic fragments, including SO_4_^−^, ZnOH^−^, and ZnO^−^, are also detected, indicating the presence of sulfate‐, hydroxide‐, and oxide‐related inorganic SEI species. The coexistence and spatial distribution of these organic and inorganic fragments provide chemical‐spatial evidence for the formation of a collagen‐derived organic–inorganic hybrid SEI. Together with the XPS depth‐profiling and Cryo‐EM results, these observations support that COL@COT promotes the formation of a thinner hybrid interphase while attenuating the accumulation of water‐induced inorganic byproducts. Previous studies have revealed that β‐sheet protein domains possess higher packing density due to their interlayer stacking configuration compared with helical structures [31]. Therefore, the relatively dense region near the Zn substrate may be associated with compact collagen‐derived domains generated during cycling, whereas the outer region may retain more hydrated and less densely packed collagen‐containing components. Nevertheless, because Cryo‐EM provides density contrast rather than direct secondary‐structure identification, and CD spectra provide ensemble‐averaged structural information without spatial resolution, the COL‐derived SEI is more cautiously described as a spatially heterogeneous collagen‐containing organic–inorganic interphase rather than a definitively resolved β‐sheet/triple‐helix bilayer.

**FIGURE 3:**
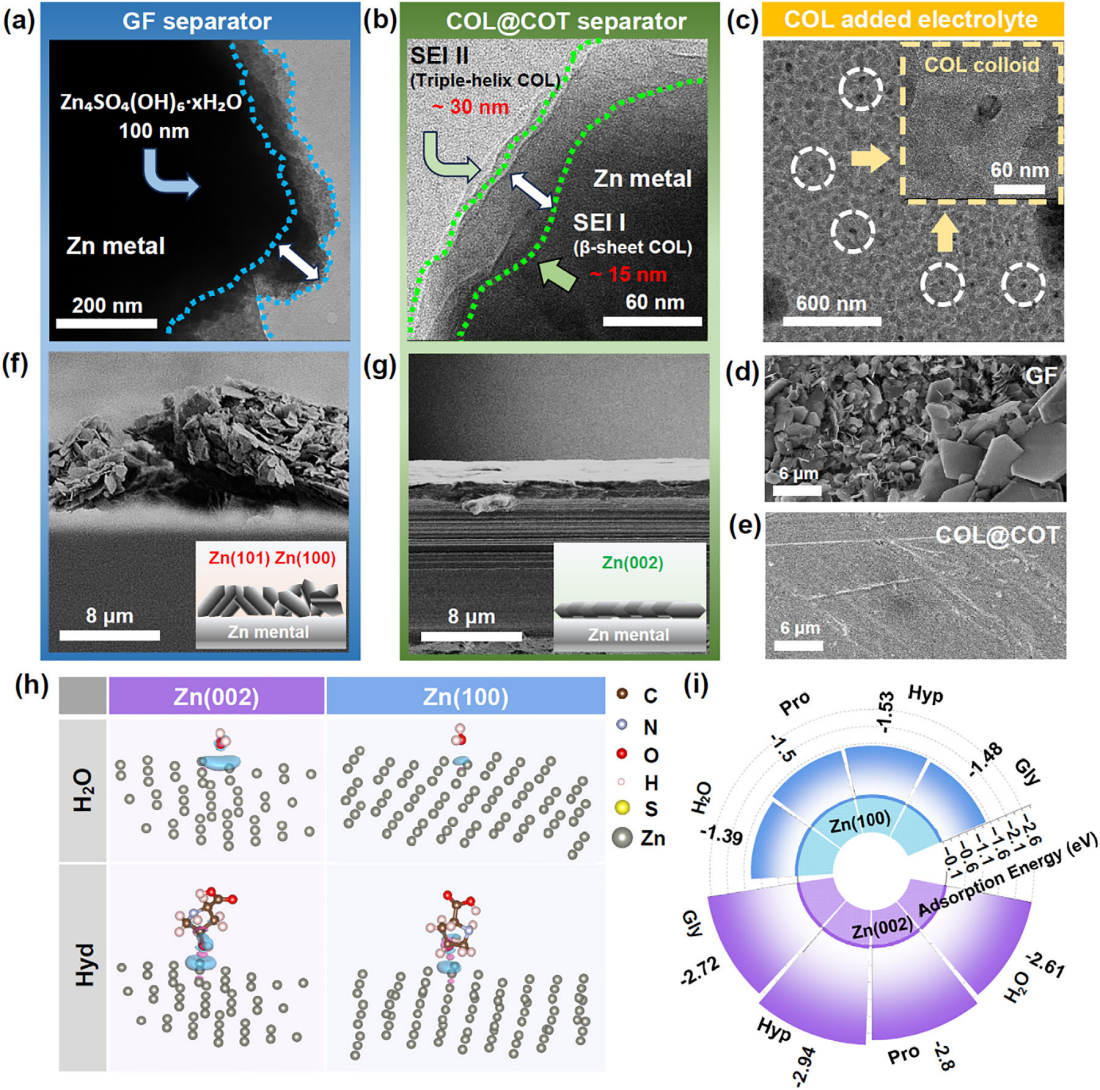
(a, b) Cryo‐EM images demonstrating morphological differences in the SEI layer on a Zn anode with (a) GF separator and (b) COL@COT separator. (c) Cryo‐EM image of electrolyte with COL@COT separator. (d–g) SEM images display (d, e) cross‐sectional and (f, g) surface morphologies of a Zn anode after cycling with the (d, g) GF separator and e, (f) COL@COT separators. (h) DFT calculations of charge distribution for the three representative COL amino acids (Gly, Pro, Hyp) and water on Zn (002) and Zn (100) planes. (i) DFT‐calculated adsorption energies of the three representative COL amino acids (Gly, Pro, Hyp) and water on different Zn planes.

To further confirm the existence form of COL molecules in the aqueous electrolyte, we employed the cryo‐sample vitrification by plunge‐freezing technique to rapidly vitrify the COL‐containing electrolyte in liquid ethane at −185°C, thereby preserving its native hydrated structure [54, 55]. The resulting ultrathin vitrified films were analyzed by cryo‐EM. As shown in Figure 3c, numerous dark, spherical micellar particles with diameters of approximately 5–15 nm were clearly observed in the vitrified electrolyte. This finding is not only consistent with the dynamic light scattering (DLS) results, further confirming the formation of 2.5‐11.9 nm micellar aggregates in the electrolyte (Figure S15), but also successfully provides direct visualization of triple‐helical macromolecules in an aqueous electrolyte, revealing that COL can spontaneously assemble into micelle‐like aggregates under electrochemical conditions. Zeta potential analysis of the electrolyte revealed that, upon the introduction of COL molecules, the surface charge of micelles shifted from negative (−4.39 mV) to positive (4.13 mV) (Figure S16). This phenomenon is consistent with the results of protein fluorescence electrophoresis, elucidating the migration direction and pathway of the COL molecules under an applied electric field.

We employed SEM to examine Zn deposition morphology after 100 cycles of plating/stripping at 1 mA cm^−2^ with a capacity limit of 1 mAh cm^−2^. As shown in Figure 3d, the GF separator led to a rough surface with disordered, aggregated Zn deposits, typical of the “dead Zn” phenomenon, which results in low Coulombic efficiency, internal short circuits, and electrolyte decomposition [56, 57]. In contrast, Zn anodes with the COL@COT separator showed smoother, denser deposits (Figure 3e). Cross‐sectional images revealed that with the GF separator, Zn grew vertically along the (100) and (101) planes, detaching as “dead Zn” (Figure 3f). With the COL@COT separator, deposition occurred mainly along the (002) plane, resulting in a more compact, smooth layer (Figure 3g). To explore the Zn deposition mechanism, we performed X‐ray diffraction (XRD) analysis. After 100 cycles, Zn anodes with GF separators showed impurity peaks (10°‐35°) in the XRD pattern (Figure S17a), while those with COL@COT separators displayed no such peaks. Zn metal's hexagonal close‐packed (HCP) structure has smoother atomic arrangements, higher density, and lower surface energy on the (002) plane compared to the (101) and (100) planes [58, 59]. The *I* (002)/*I* (100) ratio reflects deposition quality. After 90 cycles with the GF separator, this ratio decreased from 1.52 to 1.45, indicating dominant (100) plane deposition Figure S17b. For COT separators, the ratio decreased from ∼1.63 to 1.49, while with COL@COT separators, it increased from ∼1.88 to 2.65. This suggests that optimized separators promote (002) plane deposition, enhancing Zn‐ion transport and resulting in more stable, uniform deposition.

To explore the mechanism by which COL suppresses parasitic reactions in Zn cells, we investigated the adsorption behaviors of three representative amino acids in COL: glycine (Gly), proline (Pro), and hydroxyproline (Hyp) as well as water molecules on the Zn (002) plane. The calculations reveal that all three amino acids preferentially adsorb onto the Zn (002) surface rather than water molecules, suggesting that COL exhibits a stronger affinity toward Zn metal. As illustrated in Figure 3h and Figure S18, the adsorption energies and corresponding charge density difference maps were analyzed for water and the three amino acids on the Zn (002) and Zn (100) planes. The adsorption energy of COL on Zn (002) (−2.61 eV) is significantly higher than that of water, indicating a more stable interaction between COL and the Zn surface. On the Zn (100) plane, a similar trend was observed—the adsorption energy of water was lower than that of COL—further confirming the stronger interfacial binding of COL with Zn. The variation in adsorption strengths among the three amino acids originates from differences in their functional groups. As shown in Figure 3i, Gly possesses amino and carboxyl groups; Pro contains a pyrrolidine ring and a carboxyl group; while Hyp, as a hydroxylated derivative of Pro, features an additional hydroxyl group. These functional groups interact differently with Zn atoms on the (002) and (100) surfaces, leading to distinct adsorption energies. Notably, hydroxyl and carboxyl groups exhibit stronger coordination with Zn, resulting in the adsorption energy order of Hyp > Pro > Gly. Overall, the DFT results demonstrate that COL components preferentially adsorb on the Zn (002) plane rather than water molecules, thereby minimizing hydrogen evolution and other parasitic reactions during Zn deposition.

To examine the local electrical field and height variations of Zn deposition, high‐resolution KPFM was employed. It can be observed that the Zn metal anode using the conventional GF separator exhibits pronounced surface undulations of up to 1.71 µm (Figure 4a), with sharp edges clearly formed at the junctions between the (101) and (100) planes. The corresponding surface potential distribution is highly heterogeneous, with the potential intensely concentrated at the dendritic tips (marked in red). When the COT separator is used (Figure 4b), the surface roughness slightly decreases to 1.53 µm, and the potential distribution becomes marginally more uniform (green region). However, a large number of dendrites are still evident on the surface. In sharp contrast, the Zn metal anode employing the COL@COT separator displays a distinctly different morphology (Figure 4c). The surface fluctuation amplitude is significantly reduced to below 900 nm, and the deposited Zn nuclei predominantly grow along the (002) plane, forming a well‐aligned and flattened layer. Furthermore, a continuous polymeric coverage layer (COL) can be clearly observed on the surface, which effectively regulates ion flux. Most importantly, the surface potential distribution becomes remarkably uniform, and the regions of high potential intensity (red) are mainly located at the base of the Zn nuclei rather than around their peripheries, indicating a more stable and homogeneous electrochemical interface.

**FIGURE 4:**
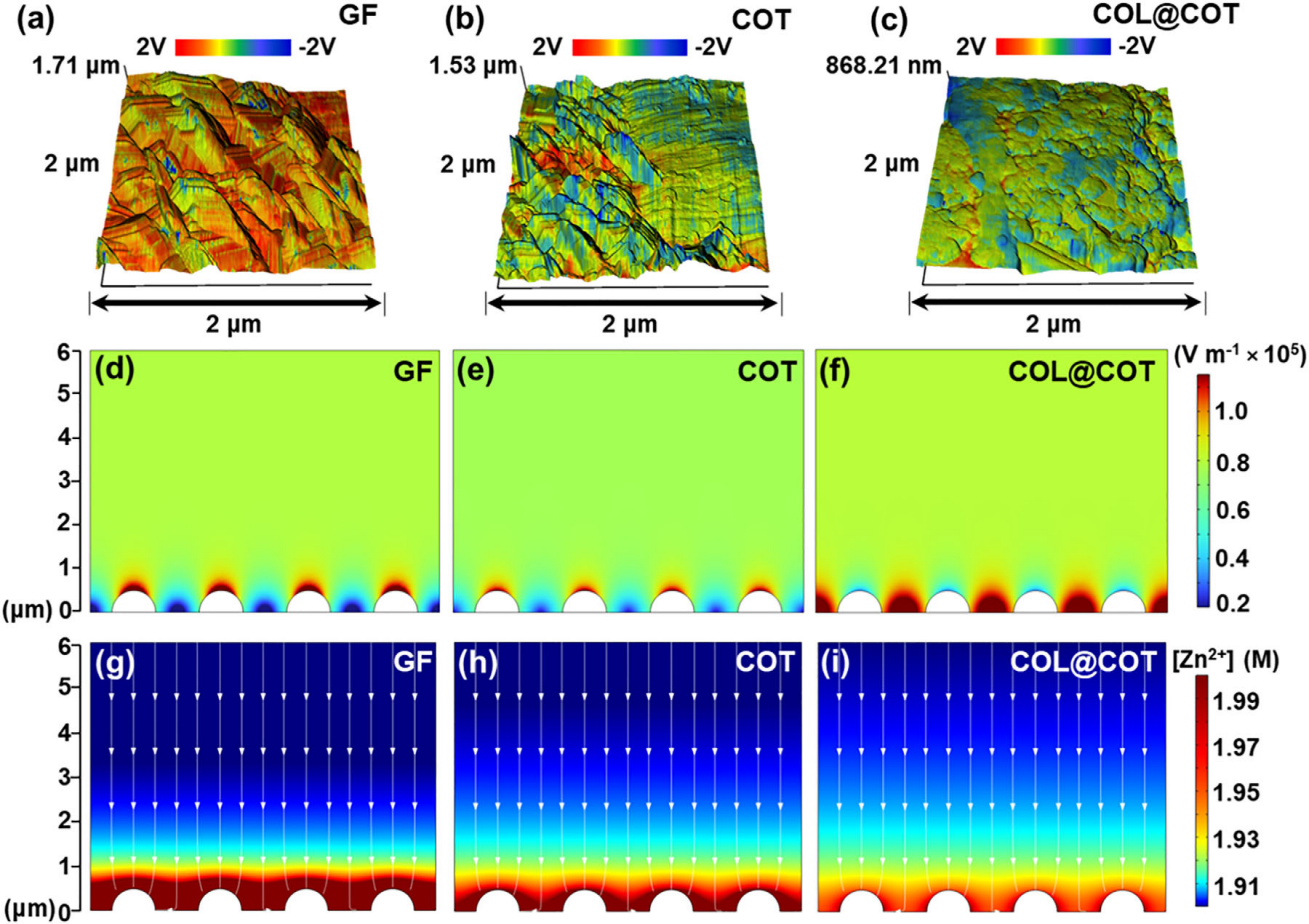
Local electric‐field and ion‐transport regulation enabled by different separators. (a–c) High‐resolution KPFM images showing the surface morphology and local potential distribution of Zn deposits formed with GF, COT, and COL@COT separators, respectively. (d–f) Finite‐element simulations (COMSOL Multiphysics) of the corresponding electric potential distribution at the Zn anode surface. (g–i) Simulated Zn^2+^ concentration distributions at the Zn anode interface under the same conditions.

Finite Element Analysis (FEA) with COMSOL Multiphysics were performed to elucidate the potential distribution and Zn^2+^ concentration profiles during Zn deposition in symmetric cells equipped with GF, COT, and COL@COT separators. As shown in Figure 4d, the GF separator induces highly uneven potential fields, with strong localization at the protruding tips of deposits, thereby accelerating dendrite growth. In contrast, the COT separator (Figure 4e) produces a relatively more homogeneous potential distribution, although distinct localized fluctuations remain. Strikingly, the COL@COT separator (Figure 4f) yields a nearly uniform potential field across the electrode surface, effectively eliminating high‐field regions prone to dendrite initiation. Consistent trends are observed in the simulated Zn^2+^ concentration distributions. With the GF separator (Figure 4g), large concentration gradients and ion depletion zones form near the deposition front, promoting nonuniform nucleation. The COT separator (Figure 4h) mitigates these gradients to some extent, whereas the COL@COT separator (Figure 4i) achieves the most homogeneous Zn^2+^ distribution, ensuring smooth ion migration and uniform Zn growth. Collectively, these results demonstrate that the COL@COT separator enables a balanced potential and ion environment at the Zn interface, thereby suppressing dendritic deposition and stabilizing the plating process.

Transparent Zn||Zn symmetric cells were employed to directly observe the Zn deposition behavior using different separators. As shown in Figure 5a, when the GF separator was used, needle‐like Zn protrusions appeared at the early stage of deposition and gradually evolved into flake‐like structures [60]. After 30 min, elongated dendrites were clearly observed, which are likely to induce the formation of “dead Zn” during subsequent stripping. In comparison, Zn deposition with the COT separator exhibited improved uniformity, although local irregularities and dendritic structures were still present [61]. Remarkably, the cell equipped with the COL@COT separator displayed dense and uniform Zn deposition without any observable dendrites, demonstrating the superior ability of the COL@COT separator to promote homogeneous Zn growth. To further evaluate the electrochemical performance of Zn anodes with different separators, linear sweep voltammetry (LSV) and Tafel analyses were conducted [62]. The Tafel plots were used to determine the corrosion potential of Zn anodes under various separator conditions. As shown in Figure 5b, corrosion potential shifted positively from approximately −0.7501 V (GF separator) and −0.7421 V (COT separator) to −0.7397 V with the COL@COT separator (vs. Ag/AgCl), indicating that the COL@COT separator effectively enhances surface passivation and mitigates corrosion. The electrochemical stability of the separators was further evaluated by LSV measurements. As shown in Figure 5c, the COL@COT separator exhibits a cathodic onset potential of −0.906 V and an anodic onset potential of 1.636 V, which are respectively shifted to more negative and more positive potentials compared with those of the GF separator (−0.864 and 1.584 V). These shifts indicate that COL@COT can suppress both hydrogen and oxygen evolution reactions, thereby improving the electrochemical compatibility of the Zn/electrolyte interface.

**FIGURE 5:**
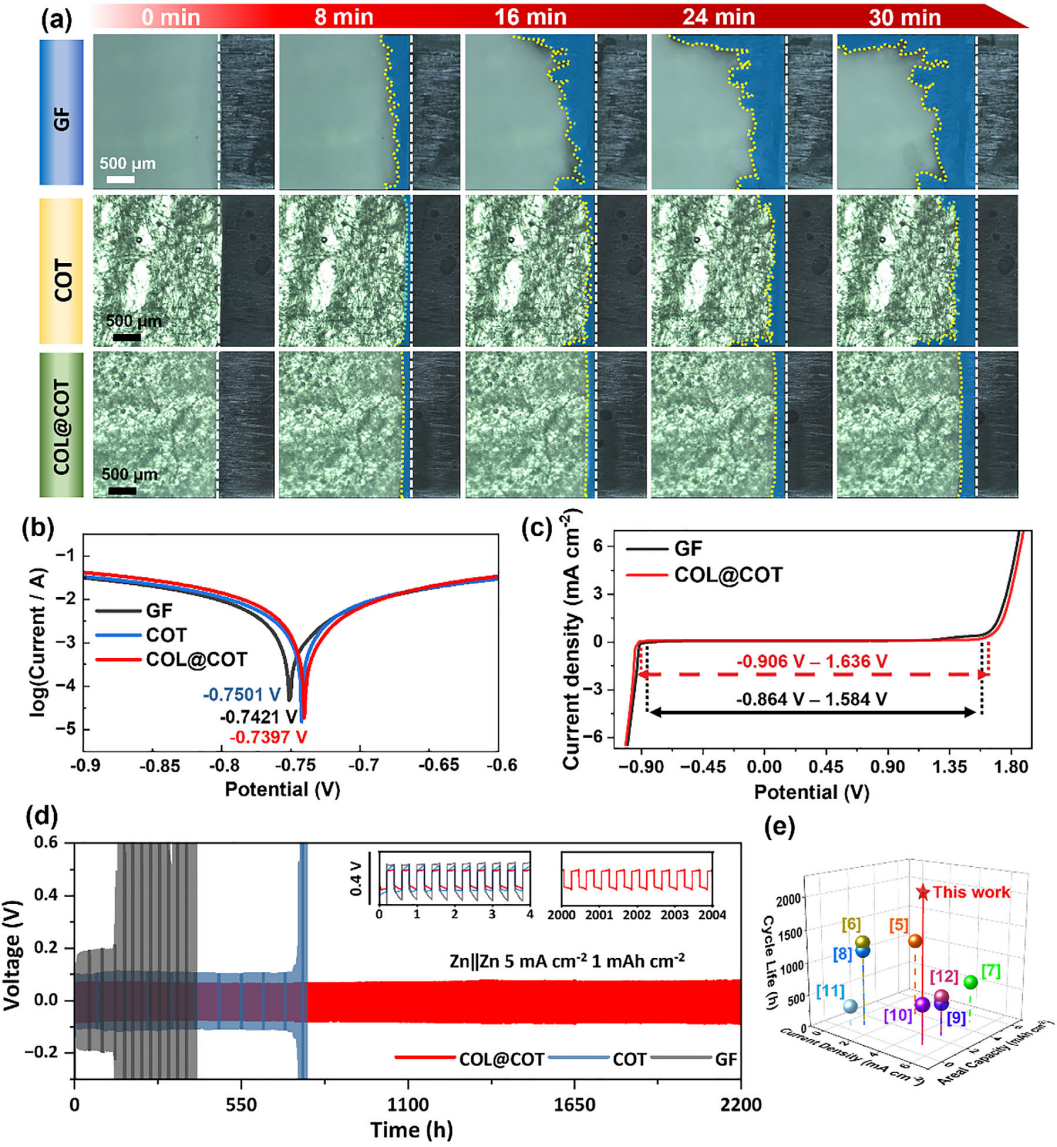
(a) In situ optical microscopy showing Zn deposition morphology changes over time at a current density of 5 mA cm^−2^ and a capacity limit of 1 mAh cm^−2^ with GF separator, COT separator and COL@COT separator. (b) Tafel corrosion curves for different separators. (c) LSV test results for different separators. (d) Long‐cycle performance of symmetric cells with different separators at a current density of 5 mA cm^−2^ and a capacity limit of 1 mAh cm^−2^. (e) Comparison of Zn||Zn symmetric cell cycle stability in this work with previously reported separators.

The long‐term stability of Zn symmetric cells was evaluated to elucidate the protective role of the COL@COT separator. As shown in Figure 5d, cells using the GF separator failed after approximately 130 h at 5 mA cm^−2^ for 1 mAh cm^−2^, whereas those with the COT separator exhibited a prolonged lifespan of around 725 h. Remarkably, cells equipped with the COL@COT separator maintained stable cycling for up to 2200 h, surpassing most previously reported Zn‐based systems (Figure 5e and Table S1). To further verify the robustness of the COL@COT separator under more demanding operating conditions, Zn||Zn symmetric cells were additionally tested at a higher current density of 10 mA cm^−^^2^ with a fixed areal capacity of 1 mAh cm^−^^2^. As shown in Figure S19, the cell using the COL@COT separator maintained a stable and smooth voltage profile over 1400 h, whereas the cells with GF and COT separators exhibited pronounced voltage fluctuations and rapid failure. These results demonstrate that the COL@COT separator can effectively stabilize the Zn/electrolyte interface even under accelerated Zn plating/stripping conditions, confirming the robust protective effect of the collagen‐regulated interphase. Throughout the entire cycling process, the COL@COT‐based cell displayed a consistently lower voltage polarization compared to the COT and GF counterparts (inset). This superior durability underscores the strong ability of the COL@COT separator to suppress dendrite formation and extend Zn anode lifetime. Furthermore, rate‐performance tests (Figure S20) confirmed that the COL@COT separator delivers stable and smooth voltage profiles even under high current densities (0.3–10 mA cm^−2^), highlighting its excellent electrochemical robustness and interfacial regulation capability.

The nucleation overpotential plays a crucial role in determining Zn deposition behavior, dendrite growth, and cycling stability. A lower overpotential generally signifies facilitated Zn nucleation and more uniform deposition. As shown in Figure 6a, the overpotential decreased progressively from ∼69.3 mV with GF separators to ∼44.6 mV with COT and further to ∼30.1 mV with COL@COT, demonstrating that the bio‐derived COL@COT interface effectively promotes smooth Zn nucleation and mitigates interfacial fluctuations. The Coulombic efficiency (CE) of Zn plating/stripping—defined as the ratio of stripped to deposited Zn per cycle—is a key metric for evaluating Zn anode reversibility and stability [63, 64]. To elucidate the influence of COL loading, Zn||Cu half‐cells were tested using separators containing varying amounts of COL. As shown in Figure 6b, increasing the COL content from 1 to 3 mg cm^−2^ markedly extended the cycling lifespan with nearly 100 % CE from ∼700 to ∼7000 cycles. However, further increasing the loading to 5 mg cm^−2^ deteriorated stability (<200 cycles), likely due to excessive Zn^2+^ chelation that impedes ion transport and enhances polarization. Hence, an optimal COL loading of 3 mg cm^−2^ achieves a balance between Zn^2+^ coordination and interfacial conductivity.

**FIGURE 6:**
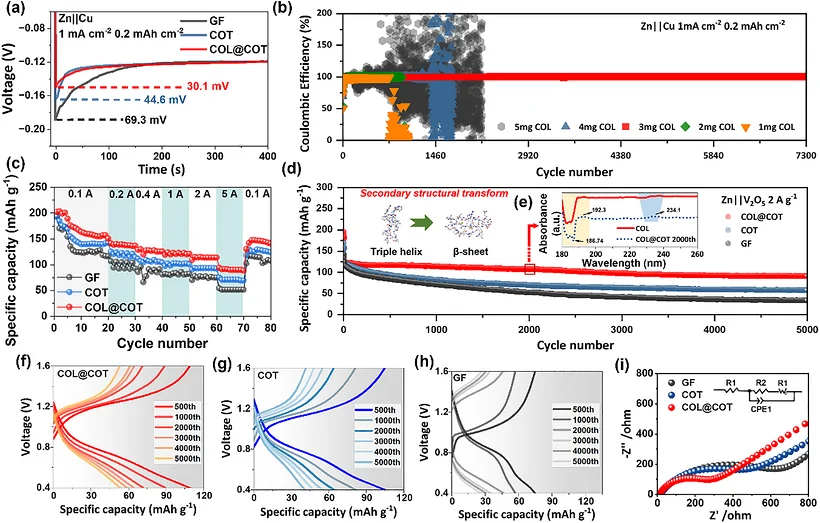
(a) Nucleation overpotential test results in Zn||Cu cells at 1 mA cm^−2^ and a capacity of 0.2 mAh cm^−2^ with different separators. (b) Long‐cycling Coulombic efficiency test results in Zn||Cu cells at 1 mA cm^−2^ and a capacity of 0.2 mAh cm^−2^ with different COL loadings. (c) Rate performance test results in Zn||V_2_O_5_ cells with different separators at current densities ranging from 0.1 to 5 A g^−1^. (d) Long‐cycle performance results in Zn||V_2_O_5_ cells with different separators at 2 A g^−1^ (first 10 cycles at 0.1 A g^−1^ for activation). (e) CD spectra of pristine collagen and that retrieved from cell after 2000 cycles. (f–h) Specific capacity–voltage curves for Zn||V_2_O_5_ cells with f) COL@COT, (g) COT, and (h) GF separators at 3 A g^−1^ for different cycles. (i) EIS plots of Zn||Zn cells using GF, COT and COL@COT separators.

The superiority of the COL@COT separator was further demonstrated in full‐cell configurations. When paired with V_2_O_5_ cathodes, the protective effect of COL on the Zn anode became evident. As shown in Figure 6c, the rate capability test revealed that the COL@COT‐based cell consistently delivered much higher and more stable capacities than those with COT or GF separators across current densities from 0.1 to 2 A g^−1^. Even at a high current density of 5 A g^−1^, the COL@COT cell retained a reversible capacity of 94.0 mAh g^−1^, outperforming the COT (74.9 mAh g^−1^) and GF (53.3 mAh g^−1^) counterparts. Notably, when the current density returned to 0.1 A g^−1^, the COL@COT‐based full cell recovered to 146.2 mAh g^−1^, showing negligible capacity fading (−6.8 % relative to the initial value), whereas the COT and GF cells declined to 128.6 mAh g^−1^ (−9.1 %) and 103.1 mAh g^−1^ (−11.2 %), respectively. Long‐term cycling at 2 A g^−1^ (Figure 6d) further confirmed the enhanced durability imparted by the COL@COT separator: after 2000 cycles, its capacity remained at 106.3 mAh g^−1^, while the COT and GF cells decayed to 68.7 and 51.3 mAh g^−1^ respectively. Notably, after 2000 cycles, the collagen species recovered from the separator–Zn interface were compared with the pristine collagen. As shown in Figure 6e, the CD spectra indicate a slight secondary‐structure variation from the triple‐helix configuration toward a β‐sheet‐like conformation. This structural evolution suggests that collagen undergoes partial rearrangement and hydrogen‐bond reorganization during prolonged cycling, enabling dynamic interfacial adaptation and preserving electrochemical stability. However, because CD spectroscopy provides ensemble‐averaged structural information without spatial resolution, these data are used to support cycling‐induced secondary‐structure variation of collagen rather than to assign spatially separated β‐sheet and triple‐helix domains within the SEI. Even after 5000 cycles at 3 A g^−^^1^ (Figure 6f–h), the COL@COT cell exhibited the lowest capacity decay, highlighting its remarkable long‐term stability. Additionally, the superior performance of the COL@COT separator was also demonstrated at moderate rates of 0.5 and 1 A g^−^^1^ (Figure S21).

Electrochemical impedance spectroscopy (EIS) was employed to gain further insights into ionic conductivity and interfacial stability. As shown in Figure 6i, the symmetric Zn||Zn cells assembled with the COL@COT separator exhibited the smallest semicircle radius, indicating the lowest charge‐transfer resistance among all samples. Based on EIS fitting, the ionic conductivity of the SEI layer formed with the COL@COT separator reached 1.21 × 10^−6^ S cm^−1^, surpassing those with GF (0.84 × 10^−6^ S cm^−1^) and COT (0.98 × 10^−6^ S cm^−1^). Interestingly, the SEI derived from the triple‐helix collagen structure exhibited ionic conductivity comparable to, or even higher than, that of previously reported SEI layers formed from α‐helical or amorphous protein conformations. This enhancement can be ascribed to the inherently ordered helical topology and directional alignment of the triple‐helix structure, which together promote efficient Zn^2+^ transport channels and facilitate rapid ionic flux across the interface.

## 3 Conclusion

In this work, we demonstrate that the intrinsic triple‐helix hierarchy of collagen can act as a biomolecular regulator to construct self‐adaptive interphases for Zn metal anodes. The COL@COT separator continuously releases collagen molecules that migrate under the electric field to assemble an organic–inorganic hybrid SEI, forming a continuous ion‐transport channel bridging the anode, electrolyte, and cathode. The ordered triple‐helix configuration, together with the Zn^2+^‐binding motifs (Gly, Pro, and Hyp), enables highly selective Zn‐ion coordination, reduces desolvation energy, and promotes (002)‐oriented Zn deposition. Meanwhile, the structural adaptability of collagen allows partial conversion of triple‐helix to β‐sheet domains during long‐term cycling, facilitating hydrogen‐bond reorganization and maintaining interfacial stability. These collective effects not only suppress dendrite formation and side reactions but also endow the biogenic SEI with remarkable mechanical resilience and ionic conductivity. Consequently, symmetric and full cells equipped with the COL@COT separator achieve ultralong cycling stability and high Coulombic efficiency, surpassing most previously reported aqueous Zn systems. Beyond performance enhancement, this study highlights a broader concept: secondary structures of biological macromolecules can be directly harnessed to regulate electrochemical interfaces. The present collagen‐inspired design establishes a prototype for developing bio‐derived and sustainable materials, in which secondary‐structure programming enables rational interphase design for next‐generation aqueous and implantable energy storage devices. The authors have cited additional references within the Supporting Information [17, 18, 19, 65, 66, 67, 68, 69, 70, 71, 72, 73, 74, 75, 76, 77, 78].

## Author Contributions

**Di He**: investigation. **Tianyi Wang**: conceptualization, methodology, data curation, investigation, validation, writing – original draft. **Yuting Qin**: investigation. **Chengyin Wang**: writing – review and editing, supervision, conceptualization, investigation, methodology, project administration, funding acquisition. **Jian Yang**: investigation, data curation. **Jiahui Lu**: methodology, data curation, investigation, formal analysis, writing – original draft. **Jianfei Shi**: investigation. **Zixia Lin**: investigation. **Guoxiu Wang**: supervision, writing – review and editing. **Bing Sun**: writing – review and editing, supervision, methodology. **Jiabao Li**: investigation, funding acquisition. **Wei Gu**: investigation.

## Conflicts of Interest

The authors declare no conflicts of interest.

## Supporting information




**Supporting File**: advs77079‐sup‐0001‐SuppMat.docx.

## Data Availability

The data that support the findings of this study are available from the corresponding author upon reasonable request.
