# Supplementary material for: Triple‐Helix Biomolecules Construct Self‐Adaptive Multi‐Phase Interfaces for Dendrites Immunized Zinc Metal Anodes
Source: Adv Sci (Weinh). 2026 Aug 12;13(61):e77079. doi: 10.1002/advs.77079 (PMC13464558; doi:10.1002/advs.77079)
Supplement: Supplementary file 1 [file ADVS-13-e77079-s001.docx]

Supporting Information

**Triple-Helix Biomolecules Construct Self-adaptive Multi-phase Interfaces for Dendrites Immunized Zinc Metal Anodes**

*Tianyi Wang ^1 †^, Jiahui Lu ^1,2 †^, Wei Gu ^1^, Jianfei Shi ^1^, Yuting Qin ^1^, Jian Yang ^3^, Di He ^1^, Zixia Lin ^4^, Jiabao Li ^1 *^, Bing Sun ^5 *^, Guoxiu Wang ^5 *^, and Chengyin Wang ^1 *^*

**Experimental Procedures**

**Materials**

1-Methyl-2-pyrrolidone (NMP) (analytical grade, ≥99%) was acquired from Sinopharm Chemical Reagent Co., Ltd. (Shanghai, P. R. China). ZnSO_4_·7H_2_O (analytical grade, 99.5%) was procured from Macklin Biochemical Co., Ltd. (Shanghai, P. R. China). V_2_O_5_ (analytical grade, ≥98%) was obtained from Sigma-Aldrich Trading Co., Ltd. (Shanghai, P. R. China). Zinc (Zn) foil (0.02 mm), copper (Cu) foil (0.01 mm), stainless steel (SS) foil (0.01 mm) were purchased from Canrd New Energy Technology Co., Ltd. (Guangdong, P. R. China). Collagen (COL) was acquired from Shanghai Macklin Biochemical Technology Co., Ltd. Cotton fiber (COT) membranes were sourced from Freudenberg Filtration Technologies GmbH & Co. KG (Jiangsu, P. R. China). Glass fiber (GF) separator membranes were purchased from Canrd New Energy Technology Co., Ltd. (Guangdong, P. R. China). Poly(vinylidene fluoride) (PVDF 900), acetylene black (Kappa 100) were obtained from Canrd New Energy Technology Co., Ltd. (Guangdong, P. R. China). The One-Step PAGE Gel Fast Preparation Kit (8%) was supplied by Nanjing Vazyme Biotech Co., Ltd. (Nanjing, P. R. China). The 180 kDa prestained protein marker was from Nanjing Vazyme Biotech Co., Ltd. (Nanjing, P. R. China). Tris-Glycine SDS-PAGE running buffer (powder) was purchased from Wuhan Servicebio Science and Technology Co., Ltd. (Wuhan, P. R. China).

**Preparation of Separators**

Glass fibers (GF) Separator: Glass fibers (GF) were cut into circles with a diameter of 19 mm using a punching machine.

Cotton fibers (COT) Separator: Cotton fibers (COT) were cut into circles with a diameter of 19 mm using a punching machine.

Collagen-coated cotton fibers (COL@COT) Separator: Collagen (COL) powder was dissolved in deionized water to prepare solutions with concentrations ranging from 5 to 25 mg/mL. The COT separators were fully immersed in the collagen solution for 5 min to ensure thorough absorption. After immersion, the separators were removed, and any excess solution was gently eliminated. The treated COT separators were then placed in a vacuum oven at 25°C and dried for 24 h. After drying, COL@COT separators with a collagen loading of 1 to 5 mg cm^-2^ were obtained.

**Material characterization**

The morphological and structural characteristics of the Zn anode were analyzed using a scanning electron microscope (SEM, S-4800). The phase structures of the Zn anode were examined through X-ray powder diffraction (XRD, Bruker-D8 ADVANCE, 5°/min). Surface morphology, elemental composition, and valence of Zn ion deposition on Cu foil were characterized using the high-resolution Kelvin Probe Force Microscopy (KPFM, SPM-9700HT, Hitachi High-Tech Corporation) and Nexsa G2 X-ray Photoelectron Spectroscopy (Thermo Fisher Scientific), respectively. XPS was conducted with a monochromatic Al target (1486.6 eV), a test beam spot size of 400 μm, full spectrum pass energy of 100 eV with a step size of 1.0 eV, narrow spectrum pass energy of 50 eV with a step size of 0.05 eV. Etching was carried out with Ar^+^ ions at 2000 eV, using SiO_2_ film as a reference standard with an etching efficiency of 0.2 nm s^-1^. The morphology of Zn ion deposition on the Cu grid was observed with a Field Emission Transmission Electron Microscope (TEM, Tecnai G2 F30 S-TWIN, FEI Company). Raman spectra were recorded using the In Via confocal Raman Spectrometer (Renishaw in Via, UK), and X-ray photoelectron spectra (XPS) were obtained using the ESCALAB 250Xi (Thermo Scientific, USA). Contact angle measurements were performed using an OCA20 Micro Contact Angle System. Adsorption features were recorded with a Varian Cary 610/670 ATR-FT-IR spectrometer in the 2000–400 cm^-1^ range. To monitor fluorescence phenomena, two-photon excitation microscopy was employed (Cari Zeiss, LSM 88-NLO), and fluorescence intensity distribution was analyzed with ZEN 3.2 software. Circular dichroism (CD) spectra were collected using a Jasco H810 spectropolarimeter, equipped with a NESLAB RTE-111 slab and purged with N_2_ gas at a flow rate of 3 to 5 ml min^-1^. The solution was placed in 0.10 cm path-length cells for detection. CD spectra were recorded from 190 to 250 nm with a resolution of 0.2 nm, accumulating five scans at a scanning rate of 100 nm min^-1^ and a response time of 0.25 s. A blank solution was measured under the same conditions, and blank levels were subtracted from the data. Western Blot Native-PAGE analysis was conducted using a Vertical Protein Electrophoresis System (Bio-Rad Laboratories, Inc.), and chemiluminescence and fluorescence imaging were performed with a ChemiScope 6000 Chemiluminescence and Fluorescence Imaging System (Shanghai Qinxing Scientific Instrument Co., Ltd.). Particle size distribution and zeta potential of various electrolytes were measured using a Malvern 3000.

**Electrochemical performance characterizations**

**Materials Preparation**

Three types of separators were used for cell assembly: GF, COT, and COL@COT. Zn||Zn symmetric cells were assembled in CR2032 coin cells using two Zn metal electrodes, 80 μL of 2 M ZnSO₄ electrolyte, and a GF, COT, or COL@COT separator. Zn||Cu half-cells were constructed using a Zn anode, Cu foil, 80 μL of aqueous electrolyte, and a GF, COT, or COL@COT separator. For Zn||V₂O₅ full cells, the cathode slurry was prepared by mixing V₂O₅ (Sigma-Aldrich Trading Co.), carbon black (CB), and polyvinylidene fluoride (PVDF) at a weight ratio of 7:2:1. The full cells were then assembled using a Zn anode, V₂O₅ cathode, aqueous electrolyte, and a GF, COT, or COL@COT separator.

The Zn||Zn symmetrical battery was assembled using two pieces of Zn metal, 80 μL of 2M ZnSO_4_ electrolyte, and a glass fibre separator in a CR2032 coin cell. The Zn||Cu half-cell was constructed with a Zn anode, Cu foil, 80 μL of aqueous electrolyte, and a glass fibre separator. For the Zn||V_2_O_5_ full cell, the cathode material, V_2_O_5_ (Sigma-Aldrich Trading Co.), was mixed with carbon black (CB) and polyvinylidene fluoride (PVDF) in a weight ratio of 7:2:1. The full cell was then assembled using the Zn anode, V_2_O_5_ cathode, aqueous electrolyte, and glass fibre separators.

**Electrochemical Testing Conditions**

All electrochemical tests were performed at 25°C. For the Coulomb efficiency test, Zn was plated onto Cu foil under a constant current, applying the required current density and capacity. After plating, Zn was stripped by charging, with a cut-off voltage of 0.5 V vs. Zn^2+^/Zn. These Zn plating/stripping tests were conducted on a Neware battery tester. For the long-term cycling stability test, the Zn||V_2_O_5_ full cells was activated by cycling at 0.1 A g^-1^ for 10 cycles within a voltage window of 0.4 to 1.6 V before testing at 5 A g^-1^.

Electrochemical impedance was measured using a CHI660E electrochemical station with a frequency range from 0.1 Hz to 100 kHz. Based on electrochemical impedance spectroscopy (EIS) results, the SEI ionic conductivity (σ) is calculated using the formula:

$\sigma=\frac{l}{R_{SEI}\cdot A}$(1)

In this equation, *σ* represents the ionic conductivity, *l* denotes the thickness of the SEI, *R_SE_*_I_ is the SEI resistance, and *A* signifies the electrode area. ^[65,66]^

**Cryogenic Electron Microscopy (Cryo-EM) characterization**

Cryo-electron microscopy (Cryo-EM) was performed using a Tecnai G2 F30 S-TWIN Field Emission Transmission Electron Microscope (FEI Company) with an accelerating voltage of 300 kV. The microscope was equipped with an aberration-corrected objective lens, which was calibrated to ensure optimal imaging conditions before each sample analysis.

For Cryo-EM sample preparation, coin cells were assembled with the working electrode attached to 300 mesh Cu transmission electron microscopy (TEM) grids. After Zn metal was deposited onto the TEM grid, the coin cells were disassembled and thoroughly washed with deionized water to remove any residual Zn salts. Following drying, the sample was prepared using the TFV method and immediately cryopreserved in liquid ethane at -185°C, resulting in an ultrathin, electron-transparent vitrified specimen.

The prepared TEM grid was mounted onto a Cryo-holder, which was then transferred through a Cryo-transfer station submerged in liquid nitrogen (LN_2_). The transfer process, lasting approximately 1 second, was carried out while the built-in shutter of the holder prevented exposure of the sample to ambient air. The Cryo-holder, containing an LN_2_ dewar bottle, ensured the sample maintained cryogenic temperatures during transfer. Throughout the characterization process, the sample was maintained at a constant temperature of -178°C.

**Western Blot (WB) characterization**: Western Blot (WB) involves gel electrophoresis under an electric field, allowing the observation of protein migration through protein bands on the gel. The procedure was as follows: First, COL@COT samples were soaked in electrophoresis buffer and loaded into the sample wells of a polyacrylamide gel. The gel was then placed in an electrophoresis chamber and subjected to 120V for 120 min. After electrophoresis, the polyacrylamide gel was stained with Coomassie Brilliant Blue to visualize the protein migration paths.

**Theoretical calculations**

Computational methods: The calculations were performed using the Gaussian and Vienna Ab initio Simulation Package (VASP) software. The structures were visualized with VESTA software. ^[67-69]^

Adsorption energy and charge difference plots: The exchange-correlation interactions of electrons were treated using the Perdew-Burke-Ernzerhof (PBE) functional within the generalized gradient approximation (GGA). The interaction between the ion core and valence electrons was modeled using Ultrasoft pseudopotentials. A kinetic energy cutoff of 500 eV was applied for the plane-wave basis set. The Brillouin zone integration employed a gamma-centered 5 × 5 × 1 Monkhorst-Pack mesh of k-points. The convergence criterion was set to 10^-5^ eV between two ionic steps for the self-consistent process, with a total energy calculation tolerance of 0.02 eV/Å.

A vacuum space of 20 Å was included in the horizontal direction to prevent interactions between the Proline (Pro), Glycine (Gly), Hydroxyproline (Hyp), and their periodic images. The adsorption energy (*E_ads_*) of H₂O or Pro/Gly/Hyp at the sites in each model was calculated as follows:

$E_{ads} = E_{Pro/Gly/Hyp/H_{2}O+ Zn(002)} - \left( E_{Pro/Gly/Hyp/H_{2}O} + E_{Zn(002)} \right)$(2)

Where the total energies of Zn(002), Pro/Gly/Hyp or H_2_O, Pro/Gly/Hyp or H_2_O with Zn(002) are denoted as *E_Zn(002)_*, *E _Pro/Gly/Hyp/H2O_*, and *E _Pro/Gly/Hyp/H2O + Zn(002)_*, respectively.

The charge transfer from Pro/Gly/Hyp or H_2_O to the difference slab of our models can be visualized by three-dimensional charge difference as follows:

${\Delta\rho= \rho}_{A/B}- \rho_{A}- \rho_{B}$(3)

Where *ρ_A/B_*, *ρ_A_*, and *ρ_B_* are the charge densities of the hybrids, the clean slab, and the Pro/Gly/Hyp or H_2_O.

Finite Element Method: Finite element simulations were conducted using COMSOL Multiphysics 6.1. The simulations employed the steady-state "Electric Currents" physics interface and the "Tertiary Current Distribution" physics interface to model the electric field and ion concentration. The ion concentration follows Fick's first law of diffusion, while the electromigration adheres to the Nernst-Planck relationship. The equations are described as follows ^[71,72]^:

$\frac{\partial c_{i}}{\partial t}=\nabla(D_{i}\nabla c_{i}+z_{i}u_{mj}Fc_{i}\nabla\varphi)$(4)

$u_{mj}=\frac{D_{i}}{RT}$(5)

where:

- $c_{i}$ is the concentration of electrolyte ions,
- $t$ is the time,
- $D_{i}$ is the diffusion coefficient of different ions in the electrolyte,
- $z_{i}$ is the charge number of different ions in the electrolyte,
- $u_{m,j}$ is the mobility,
- *F* is the Faraday constant,
- *φ* is the electric potential,
- $R$ is the gas constant,
- *T* is the absolute temperature.

The analysis involving electrode kinetics further introduces the Butler-Volmer equation for solving ^[73]^:

$i_{loc}=i_{0}(exp\left( \frac{\alpha F_{\eta}}{RT} \right)-exp\left( \frac{-\left( 1-\alpha\right)F_{\eta}}{RT} \right))$(6)

where:

- $i_{loc}$ is the local current density at the electrode,
- $i_{0}$ is the exchange current density,
- $\alpha$ is the anodic transfer coefficient,
- $\eta$ is the overpotential.

Both models consider a simplified surface structure of the Zn anode, with semicircular protrusions representing the initial dendrite formation centers during Zn deposition. The simulation primarily solves for the electrolyte. The boundary conditions are set by the potentials of the cathode and anode, with the cathode potential set to zero and the anode potential set to the polarization voltage measured in a Zn symmetric cell experiment. The initial Zn^2+^ concentration is set to 2 mol/L, and its diffusion coefficient in the electrolyte is set to 2 × 10^−9^ m^2^/s. The ionic conductivities for the GF, COT, and COL@COT models are 0.84 × 10^−6^ S cm^-1^, 0.98 × 10^−6^ S cm^-1^ and 1.21 × 10^−6^ S cm^-1^, respectively. The exchange current density for the battery is set to 20 mA cm^-2^, and the system temperature is set to 298 K.

**Figure S1.** Digital photos and cost comparison of different separators: a) Cost per unit area of GF, COT, and COL@COT. b) Unpunched COT separator. c-e) Digital photos of COT, COL@COT, and COL@COT with 2M ZnSO_4_, respectively.

**Figure S2.** SEM images of different separators: a-c) GF, e-g) COT, i-k) COL@COT. Corresponding fiber diameter distributions based on SEM analysis: d) GF, h) COT, l) COL@COT.

**Figure S3.** SEM images of different separators at varying magnifications: a,d) GF; b,e) COT; c,f) COL@COT. a-c) High magnification images; d-f) Low magnification images.

**Figure S4.** Contact angle measurements of different separators with 2M ZnSO_4_ electrolyte: a-b) COL@COT, c-d) COT, e-f) GF.

**Figure S5.** KPFM topographic images of a) COL and b) COL@COT. Compared with pristine COL, COL@COT exhibits a smoother and more continuous surface morphology, indicating the formation of a homogeneous collagen-modified fiber surface.

**Figure S6.** Two-photon confocal microscopy images of COL@COT and COT separators: a, c) 2D images of COL@COT and COT separators, respectively. b, d) Corresponding 2.5D images of COL@COT and COT separators.

**Figure S7.** CD spectra of pristine COL and that retrieved from COL@COT separator.

**Figure S8.** Two-photon confocal microscopy image of fluorescent distribution around the edge and protrusions on a Zn metal foil using different separators under UV light.

**Figure S9.** RDFs g(r) of a) Zn-S and b) Zn-O pairs with H_2_O, Gly, Hyp and Pro.

**Figure S10.** Optimized structures of the solvent, (Zn-Solvent)^2+^ and (Zn-Solvent_2_)^2+^ for H_2_O, Gly, Hyp and Pro.

**Figure S11.** XPS spectra of C 1s for the SEI formed in Zn||Cu cells using a COL@COT as separator.

**Figure S12.** XPS spectra of N 1s for the SEI formed in Zn||Cu cells using a COL@COT as separator.

**Figure S13.** XPS spectra of Zn 2p for the SEI formed in Zn||Cu cells using a COL@COT as separator.

**Figure S14.** TOF-SIMS three-dimensional reconstruction of CN⁻, CNO⁻, C₄H₈NO⁺, SO₄⁻, ZnOH⁻, and ZnO⁻ fragments in the interfacial layer of the cycled Zn electrode with the COL@COT separator.

**Figure S15.** The particle size distribution of 2M ZnSO_4_ with and without a COL@COT.

**Figure S16.** The Zeta potential of 2M ZnSO_4_ with and without a COL@COT.

**Figure S17.** a) XRD patterns of Zn anodes retrieved from Zn||Zn symmetric cells with different separators after cycling. b) The corresponding *I*_(002)_/*I*_(100)_ ratio.

**Figure S18.** The adsorption energies and charge difference of Zn (002) and Zn (100) with Pro and Gly.

**Table S1.** Assessment of cycling stability in Zn||Zn symmetrical cells relative to previously reported separators.

| **Separator Material** | **Current Density (mA cm^-2^)** | **Areal Capacity (mAh cm^-2^)** | **Cycle Life (h)** | **Journal** | **Ref.** |
| --- | --- | --- | --- | --- | --- |
| CF-SO_3_ | 2 | 4 | 1200 | Adv. Funct. Mater. | [74] |
| PTFE | 1 | 1 | 1300 | Adv. Funct. Mater. | [19] |
| Zr-CNF | 5 | 5 | 640 | Adv. Funct. Mater. | [75] |
| MXene-GF | 1 | 1 | 1180 | Adv. Funct. Mater. | [18] |
| Cellulose | 5 | 2.5 | 500 | Adv. Energy Mater. | [76] |
| Modification Cellulose | 5 | 1 | 600 | Adv. Energy Mater. | [77] |
| Janus separator | 0.5 | 0.5 | 300 | Adv. Mater. | [78] |
| C/Cu-700 | 5 | 2.5 | 600 | Adv. Mater. | [17] |
| COL@COT | 5 | 1 | 2200 | - | **This work** |

**Figure S19.** Cycling performance of Zn||Zn symmetric cells using GF, COT, and COL@COT separators at 10 mA cm⁻² and 1 mAh cm⁻².

**Figure S20.** Cycling stability of Zn||Zn cells using different separators under various current densities.

**Figure S21.** The electrochemical cycling performance of the assembled full cells with different separators at a) 0.5 A g^-1^ and b) 1 A g^-1^ current densities.
